# The mechanism underlying metastasis in triple-negative breast cancer: focusing on the interplay between ferroptosis, epithelial-mesenchymal transition, and non-coding RNAs

**DOI:** 10.3389/fphar.2024.1437022

**Published:** 2025-01-15

**Authors:** Ziyi Chen, Yi Zhao

**Affiliations:** ^1^ Institute for Translational Medicine, The Affiliated Hospital of Qingdao University, College of Medicine, Qingdao University, Qingdao, China; ^2^ Key Laboratory of Birth Regulation and Control Technology of National Health Commission of China, Maternal and Child Healthcare Hospital of Shandong Province Affiliated to Qingdao University, Jinan, Shandong, China

**Keywords:** ferroptosis, EMT, breast cancer, non-coding RNA, metastasis

## Abstract

Triple-negative breast cancer (TNBC) is a type of breast cancer with lack the expression of estrogen receptor (ER), progesterone receptor (PR), and human epidermal growth factor receptor 2 (HER2). It is the most aggressive breast cancer and the most difficult to treat due to its poor response to treatments and extremely invasive characteristics. The typical treatment for TNBC frequently results in relapse because of the lack of particular treatment choices. It is urgent to focus on identifying a workable and effective target for the treatment of TNBC. Cancer metastasis is significantly influenced by epithelial-mesenchymal transition (EMT). Ferroptosis is an iron-dependent cell death form, and changes its key factor to affect the proliferation and metastasis of TNBC. Several reports have established associations between EMT and ferroptosis in TNBC metastasis. Furthermore, non-coding RNA (ncRNA), which has been previously described, can also control cancer cell death and metastasis. Thus, in this review, we summarize the correlation and pathways among the ferroptosis, EMT, and ncRNAs in TNBC metastasis. Also, aim to find out a novel strategy for TNBC treatment through the ncRNA-ferroptosis-EMT axis.

## 1 Introduction

Breast cancer (BC) remains the most prevalent gynecological malignancy worldwide (Harbeck and Gnant, 2017). Based on the expression of molecular markers, estrogen receptor (ER), progesterone receptor (PR), and human epidermal growth factor receptor 2 (HER2), BC can be categorized into three subtypes: luminal-like (hormone receptor (HR)+/HER2-), HER2 positive (HER2+), and triple-negative (HR-, HER2-) breast cancer (Yeo and Guan, 2017; Barzaman et al., 2020). Triple-negative breast cancer (TNBC) is distinguished by the absence of ER, PR, and HER2 expression, which makes up around 15% of all breast cancer. Subsequently, a study analyzed the gene expression profiles of 587 TNBC patients and classified TNBC into six subtypes, which were basal-like 1 (BL1), basal-like 2 (BL2), luminal androgen receptor (LAR), immunomodulatory (IM), mesenchymal (M), and mesenchymal stem-like (MSL) (Lehmann et al., 2011). Due to differences in gene expression, each TNBC subtype is differently sensitive to treatment. For instance, the M subtype is susceptible to resistance to chemotherapeutic agents, and the LAR subtype is more effectively treated with anti-AR therapy (Yin et al., 2020). Thus, TNBC now is recognized as a refractory illness due to the lack of well-defined molecular targets (Lehmann et al., 2011; Jin et al., 2021). The manifestations of TNBC include increased angiogenesis and epithelial-mesenchymal transition (EMT), and it has more invasive clinical features compared with other two types of breast cancer (Bao et al., 2021). Moreover, TNBC is more likely to relapse and its overall survival rate is shorter than other types of breast cancer (Dastjerd et al., 2022). Unfortunately, current therapies, like radiation, immunotherapy, and chemotherapy, are not effective due to their serious side effects and multidrug resistance (Dastjerd et al., 2022; Ben-Dror et al., 2022; Kaur and Jaitak, 2019). Therefore, investigation of the novel targets for TNBC treatment becomes urgent issues.

Ferroptosis is a brand-new type of cell death that has been identified as an iron-caused and reactive oxygen species (ROS)-dependent cell death (Mou et al., 2019). In contrast to atypical apoptosis and necrosis, the characteristics of ferroptosis are mainly a decrease in cell volume and an augmentation of mitochondrial membrane density (Yu et al., 2017). Because of the presence of oxidative stress and severe membrane lipid peroxidation, plasma membrane loses the selective permeability, then resulting the above changes in cell morphology (Mou et al., 2019). Ferroptosis is caused by either restricting cysteine absorption or inactivating glutathione peroxidase 4 (GPX4), thus creating an accumulation of ROS (Latunde-Dada, 2017). Currently, ferroptosis mechanisms in cancers have become a hot topic. The latest progress shows that the three main pathways of regulating cellular to ferroptosis are the GSH-GPX4, GCH1-BH4 and NADPH-FAP1-CoQ10 (Wang H. et al., 2021). More and more evidences indicate that ferroptosis could inhibit tumor cell growth and development, therefore, targeting ferroptosis might be an effective treatment of cancer (Li Z. et al., 2020).

Cancer metastasis is regarded as a process of concurrence and partially reduplication, and play a critical role in cancer death (Suhail et al., 2019). The potential of primary malignancies to spread to neighboring tissue and far-off organs is noteworthy with breast tumors primary migrating to bone, lungs, liver, and brain, then tumor recurrence is caused in corresponding sites (Howard et al., 2022; Nguyen et al., 2009). Notably, extensive evidences have been presented that EMT program is a key factor in tumor metastasis (Williams et al., 2019; Park et al., 2022). Moreover, it is believed that EMT is a factor in the spread of various tumors, including breast, colorectal, ovarian, pancreatic, prostate and renal (Yao J. et al., 2021; Lambert et al., 2017). This morphogenetic transition, which causes epithelial cells to acquire mesenchymal characteristics, is associated with a variety of tumor functions, such as tumor initiation, invasiveness, metastasis, and chemoresistance of malignant tumors, as well as tumor stemness (Babaei et al., 2021; Pan et al., 2021; Pastushenko and Blanpain, 2019).

According to the Human Genome Project, the vast majority of the human genome is made up of non-protein-coding regions, which were largely transcribed into non-coding RNAs (ncRNAs) (Xu et al., 2020). Non-coding RNA ordinarily refers to RNA that does not encode protein, but contains information or has some functions (Mattick and Makunin, 2006). Based on their length, non-coding RNA approximately can be split into long non-coding RNAs (lncRNAs) and short non-coding RNAs (sncRNAs), and the latter include small interfering RNA (siRNAs), small nucleolar RNAs (snoRNAs), microRNAs (miRNAs), PIWI-interacting RNAs (piRNAs), and so on (Xu et al., 2020). miRNA, lncRNA, circRNA, and piRNA were major types of ncRNA, and they played various functions among cancers (Yan and Bu, 2021). Moreover, as oncogenes or suppressor genes, non-coding RNAs could reduce or suppress the incidence and development of TNBC (Liu J. et al., 2021). Previous researches show the expression of miRNA related to TNBC cells metastasis, and EMT was regarded as the main mechanism of cancer cell metastasis. Take miR-125b for an example, its lower expression in TNBC cells is accompanied by the decrease of cell migration and invasion (Xu et al., 2020). Of note, ncRNAs regulate the potential mechanism of ferroptosis, though mitochondrial-related proteins and peroxidation of lipid, iron or glutathione. More and more studies have shown that the role of non-coding RNA regulation breast cancer progression via ferroptosis, and presumably coupled with EMT (Zuo et al., 2022). In this review, we aim to summarize the relationship between ferroptosis and EMT in TNBC progression, highlight the significance of ncRNAs in the development of TNBC, and suggest prospective therapeutic approaches for TNBC treatment.

## 2 Ferroptosis in TNBC metastasis

Nowadays, more and more studies present TNBC behaves susceptibility to ferroptosis, and highlight the pathway as a treatment of TNBC (Verma et al., 2020; Sun L. L. et al., 2021; Doll et al., 2017). In 2012, Dixon proposed the concept of ‘Ferroptosis’ to explain a cell death process reliant on iron. They also confirmed that the small molecule ferrostatin-1 (Fer-1) acts as a telling ferroptosis inhibitor. Fer-1 prevents the accumulation of ROS in cytoplasm and lipid induced by erastin and shows no inhibition in the MEK/ERK pathway, chelated iron and synthesis of protein. Erastin restrains the assimilation of cystine by cystine/glutamic acid antiporter (system XC-), which destroys the antioxidant defense of cells and eventually leads to iron-dependent oxidative death (Dixon et al., 2012). Then 10 years after the concept of ferroptosis was put forward, the lab systematically concluded the key discoveries associated with ferroptosis during the time. In 2014, they reported GPX4 as an important inhibitor to ferroptotic cancer cell death (Stockwell, 2022). The inactivation of GPXs, when treated with erastin or BSO, leads to the buildup of ROS in both cytoplasm and lipid. It was also found that GPX4 modulates ferroptosis induced by 12 diverse compounds (Yang et al., 2014). Moreover, p53, ACSL4, and LPCAT3 were corroborated as promoters to ferroptosis (Stockwell, 2022). Subsequently, several discoveries, such as ALOXs, CD8^+^ T cells, FSP1-CoQ10, GCH1/BH4, radiation, and DHODH, related to ferroptosis were successively reported (Stockwell, 2022).

Dixon’s research uncovered a divergence between erastin-induced cell death and apoptosis, necrosis and other types of cell death, with distinct morphological, biochemical and genetic distinctions. From the morphological point of view, mitochondria shrink, membrane density increases and mitochondrial cristae diminishes or vanishes (Dixon et al., 2012). Nonetheless, the cell membrane maintains integrated, and the size of nucleus is regular (Dixon et al., 2012; Li J. et al., 2020). Biochemically, ferroptosis is mainly reflected in iron accumulation and lipid peroxidation. On the one hand, iron produces redundant ROS directly by Fenton reaction, thus aggravating oxidative damage. On the other hand, iron actives lipoxygenase (ALOX) or EGLN prolyl hydroxylases to regulate lipid peroxidation and oxygen homeostasis indirectly (Tang et al., 2021). In genetics, a group of diverse genes modulate ferroptosis (Dixon et al., 2012). Taking PTGS2 (a gene encoding cuclooxygenase-2) as example, upregulation of PTGS2 is regarded as a downstream maker of ferroptosis (Yang et al., 2014). Furthermore, the genes related to GSH system, Coenzyme Q10 (CoQ10) system, NFE2L2 transcription pathway and other antioxidant defenses or connected with membrane repair (e.g., ESCRT-III pathway) effectively control the membrane damage during ferroptosis (Tang et al., 2021).

Nowadays, various studies have given the evidence that inductive ferroptosis has promising prospects in the treatment of cancer and the characteristic of cell metastasis is resistance to ferroptosis. It is also proved that tumors with resistance to traditional therapy or highly metastasis tendency show more sensitivity to ferroptosis induction (Liu W. et al., 2021; Jiang et al., 2023; Lu et al., 2017). Interestingly, TNBC exhibited ferroptosis heterogeneity. GPX4 inhibitors might be used to treat luminal androgen receptor (LAR)-subtype TNBC through androgen receptor (AR) signaling pathway (Jiang et al., 2023). Theoretically, we might discover the correlation between ferroptosis and TNBC treatment, through a comprehensive and deepgoing retrospective analysis.

**FIGURE 1:**
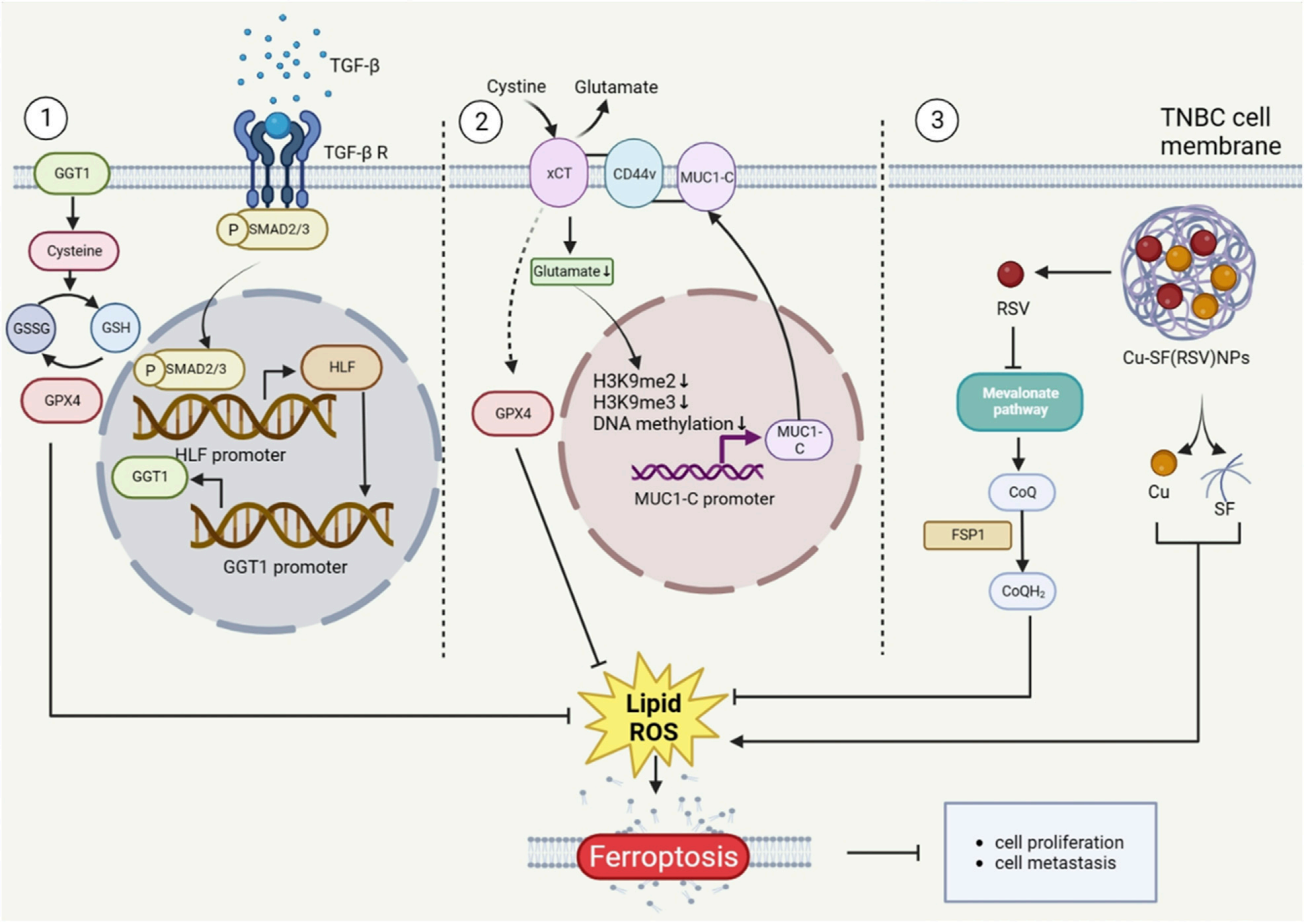
The Role of ferroptosis in TNBC metastasis. The proteins that TNBC cells make play a function in ferroptosis regulation, which controls the proliferation of TNBC cells. Additionally, Cu-SF(RSV) NPs operate on the CoQ10 mechanism that controls ferroptosis. The figure was created using BioRender.com.

Hepatic leukemia factor (HLF), an innovative oncoprotein, enhanced ferroptosis resistance by transactivating gamma-glutamyltransferase 1 (GGT1), then prompting proliferation and metastasis of TNBC (Li H. et al., 2022). Additionally, MUC1-C/xCT axis inhibits ferroptosis and then contributes to the survival rate of TNBC cells. Erastin, on the other hand, regulates the mechanism that causes TNBC ferropotic cell death (Hasegawa et al., 2016). Meanwhile, other study has also shown that erastin united with anti-CD24 antibody could restrain the growth of tumor and enhance the animal survival rate (Hou et al., 2022). Several studies proved that nanoparticles, which can alleviate the drug resistance, were used to the treatment of TNBC by inducing ferroptosis (Chen et al., 2023; Zhang J. et al., 2021). For example, Yang et al. highlight that rosuvastatin (RSV) wrapped in silk fibroin (SF) nanoparticle, also was called Cu-SF(RSV) NPs, inhibits TNBC by conquering FPS1-medicated ferroptosis resistance. Moreover, RSV could hinder neoplasm metastasis through lowering the expression of MMP9, which is simulated by tumor derivative factors. Particularly, it was found that Cu-SF(RSV) NPs efficiently inhibited 4T1 tumor metastasis, and included in the consideration of assistant drugs for breast cancer (Yang et al., 2022). Through the above examples, it is possible that ferroptosis-related pathways or regulators can control the proliferation and metastasis of TNBC cells (Figure 1).

## 3 EMT in TNBC progression

**FIGURE 2:**
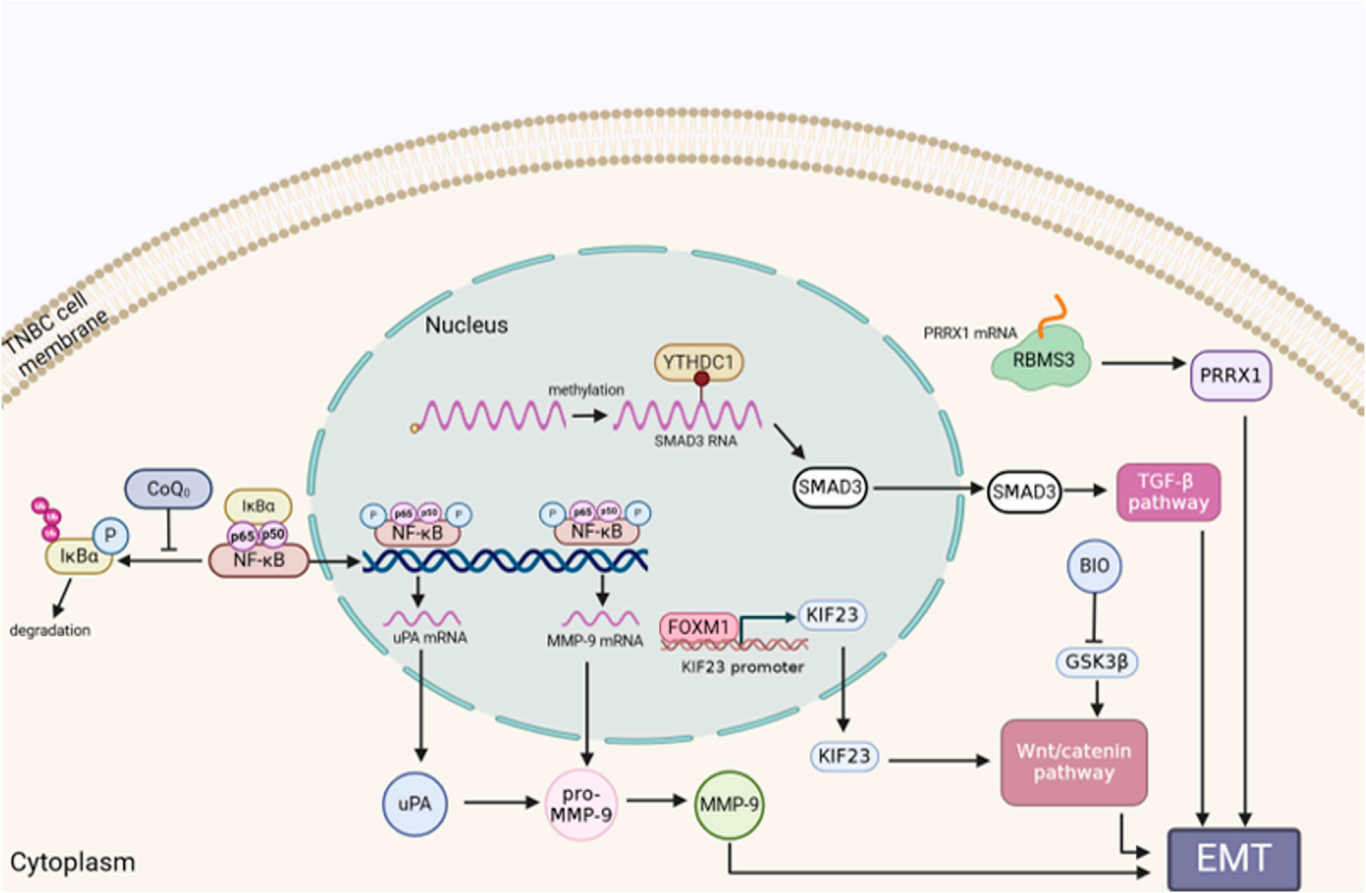
The role of EMT in TNBC metastasis. EMT is a factor in the migration, invasion, and development of TNBC cells. Genes or proteins that control EMT are correlated with the appropriate signaling pathway. The figure was created using BioRender.com.

Regarded as one of the most essential components for tumor metastasis, the epithelial-to-mesenchymal transition (EMT) is distinguished with a process of diminishing polarity and intercellular adhesion, while augmenting the capacity of migration and invasion (Yao J. et al., 2021). EMT medicated tumor metastasis under the control of transcriptions factors, such as TWIST1, SNAI1, and ZEB1 (Chen et al., 2021a; van Staalduinen et al., 2018). The latest evidence has revealed that over 90% of TNBC patients died as a result of cancer cells migrating from the primary tumor to distant organs (Yang et al., 2022). Recent study shows that the majority of TNBC cells with the CD24high phenotype expressed less E-cadherin and more vimentin and N-cadherin. This also proves that TNBC metastasis is surely accompanied by EMT program (Hou et al., 2022). Furthermore, abnormal activation of EMT in TNBC could increase the invasion, progression and recurrence ratio of tumor (Bao et al., 2021). Coenzyme Q0 (CoQ0), with high oxidation resistance, inhibits EMT via activating E-cadherin via the Wnt/β-catenin pathway and the NFκB pathway (Yang et al., 2019). Evidences show that Wnt/β-catenin pathway participates in proliferation and metastasis of TNBC. Moreover, Wnt/β-catenin can be activated by the KIF23, thereby promoting EMT in TNBC. Downregulation of KIF23 possibly influencing the mitotic function of TNBC cells (Li Z. et al., 2022). RNA N6-methyladenosine (m6A) reader YTHDC1 can enhance TGF-β-mediated EMT and improve the survival rate of TNBC cells by enhancing the nuclear output of SMAD3 mRNA (target RNA of YTHDC1) (Tan et al., 2022). Mesenchymal and stem cell-like cells make up a significant component of TNBC cells. GSK3β inhibitor is identified as a small molecule inhibitor for inhibiting EMT, and it is also capable of selectively inhibiting mesenchymal and stem cells while maintaining cells with epithelial features. Additionally, GSK3β is known as the only target associated with TNBC patient prognosis in the Wnt pathway (Vijay et al., 2019). The inhibition of tumor metastasis by RAB1B was discovered to be drastically reduced in TNBC. Lack of RAB1B could reduce the degradation of ubiquitin and increase the phosphorylation of SMAD3, thus highly expressing the protein levels of TGF-β receptor 1 (TβR1) and activating TGF-β signal pathway. Additionally, RAB1B can trigger TGF-β-induced EMT, and increase the proliferation and migration of TNBC cells both *in vitro* and *in vivo* (Jiang et al., 2015). Studies have demonstrated that MUC1-C causes EMT in breast cancer stem cell (CSC), via acting on SOX2, KLF4 and OCT4 to induce the dedifferentiation of TNBC (Kufe, 2020; Yamashita and Kufe, 2022). Moreover, the mesenchymal TNBC cell lines have increased expression of RNA binding motif single stranded interacting protein 3 (RBMS3), which controls EMT in TNBC (Block et al., 2021). Previous research proved that TNBC cells express more programmed death ligand 1 (PD-L1) than the other two types of breast cancer. Chen et al. demonstrated that PD-L1 induces EMT in TNBC cells through protecting EMT transcription factor snail from being distrusted. Mechanically, PD-L1 could activate p38-MAPK and inhibit GSK3β signalling pathway, thus preventing the phosphorylation, ubiquitination, and degradation of snail (Chen C. et al., 2021) (Figure 2).

## 4 Cross-talk between EMT and ferroptosis in TNBC metastasis

**FIGURE 3:**
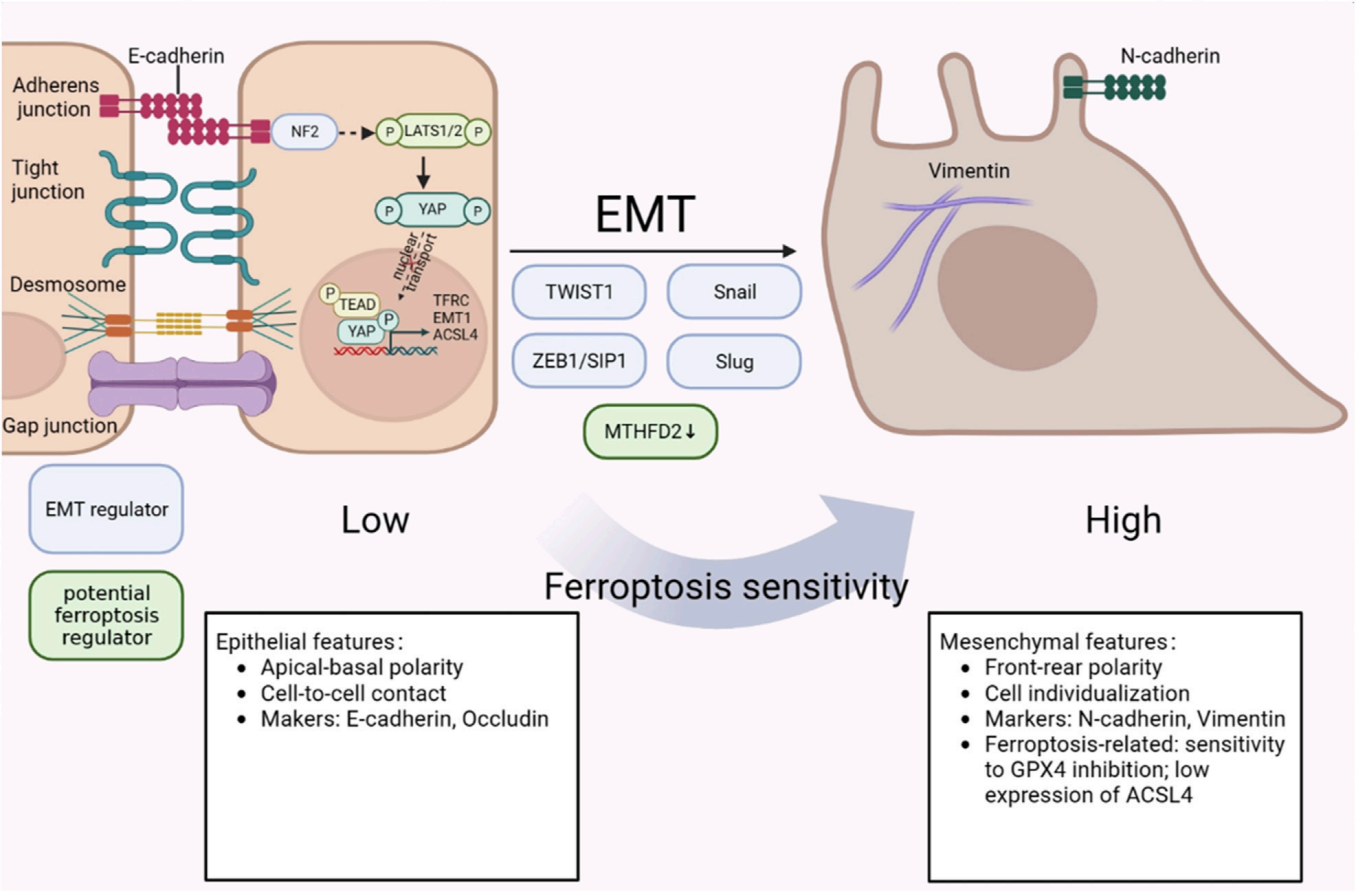
The crosstalk between ferroptosis and EMT in TNBC metastasis. Mesenchymal cells have higher ferroptosis susceptibility than epithelial cells, which are less sensitive to it. NF2-YAP pathway exhibits an inhibitory effect on ferroptosis regulators. In addition, HLF, which is regulated by TGF-β, controls ferroptosis sensitivity by targeting GGT1. The figure was created using BioRender.com.

Previous research has proved that mesenchymal-high cancers behave intensive sensibility to ferroptosis inducers, particularly GPX4 inhibitors (Bi et al., 2019). Besides, as a positive EMT regulator, protein LYRIC could contribute to ferroptosis through restraining the expression of GPX4 and SLC3A2 (Chen X. et al., 2021). The abundance of DDR2 expression activated by EMT in recurrent breast cancer cells, resulting in a heightened susceptibility to ferroptosis has been noted (Lin et al., 2021). However, the relationship between ferroptosis and EMT in triple negative breast cancer progression remains largely unexplored. Ferroptosis and EMT are the two crucial procedures and closely contact reciprocally in tumor cells (Yao J. et al., 2021). As reported, mesenchymal cancer cells are sensitive to ferroptosis, while cancer cells with EMT or metastasis tendencies are more vulnerable to ferroptosis (Wu et al., 2019). A prior study demonstrated that CSPP1 may influence ferroptosis via controlling EMT, stromal, and immunological responses in low-grade gliomas (LGG) in the brain (Wang W. et al., 2022). Additionally, it has been shown that EMT pathways can give positive feedback to ferroptosis (Chen X. et al., 2021). The increase of CD44-mediated hyaluronate-dependent iron endocytosis enhances the expression of EMT-related genes, which makes MDA-MB-468 cells more sensitive to ferroptosis (Chen X. et al., 2021; Müller et al., 2020). Hepatic leukemia factor (HLF), which acts as a novel oncoprotein in TNBC, enhanced ferroptosis resistance by transactivating gamma-glutamyl transferase 1 (GGT1), then prompting the proliferation and metastasis of TNBC (Li H. et al., 2022). However, HLF was regulated by transforming growth factor-beta 1 (TGF-β1), which is ascertained as an inducer of EMT and trigger ferroptosis in isolated breast cancer cells (Li H. et al., 2022; Wu et al., 2019; Yang et al., 2020). In Wu et al.’s study, they demonstrated that the intracellular Merlin-Hippo route, which is regulated by E-cadherin, can decrease ferroptosis in epithelial cells (Wu et al., 2019). Taken together, the above findings suggested that EMT acts as a regulator to enhance ferroptosis sensitivity. It should be noted that ferroptosis might have a positive effect on EMT program. Yang et al.'s work revealed that the markers GPX4, SCP2, and CAV1 of ferroptosis also influence the EMT features (Yao J. et al., 2021). In panc-1 CSCs, knockdown or overexpression of GPX4 can modulate EMT by upregulating E-cadherin and downregulating vimentin, Slug and Snail expression at the mRNA and protein levels (Peng et al., 2019) (Figure 3). Based on these studies, we hypothesize that there is a mutual regulation between ferroptosis and EMT. Since there is little research being conducted in the background of TNBC cells, with a focus on causing TNBC metastasis, whether EMT-regulated ferroptosis or ferroptosis-regulated EMT predominates still needs to be done in the future.

## 5 Non-coding RNA through EMT and ferroptosis features to promote TNBC metastasis

More and more evidences showed that non-coding RNA (ncRNA) is closely related to the cancer progression and ncRNAs can serve as regulators to play a role in tumor development process, such as tumor invasion and metastasis (Yan and Bu, 2021; Sakamoto et al., 2017). As reported, there are two possible modes to interpret the relationship between non-coding RNA, especially miRNA, and the development of tumor. One is that the expression of neoplastic suppressor genes might be suppressed by the upregulation of miRNA, thus promoting tumorigenesis. Another is that the expression of associated oncogenes might be promoted by the downregulation of miRNA, thus similarly triggering tumorigenesis (Duan et al., 2021; Caldas and Brenton, 2005). For example, the depletion of circRHOT1 enhanced erastin-induced growth inhibition in MDA-MB-231 TNBC cells. When circRHOT1 expression is inhibited, Fe and ROS expression levels are elevated and GPX4 and SLC7A11 expression is weakened. In their study, they highlighted that circRHOT1 sponged miR-106a-5p to inhibit ferroptosis in breast cancer (Zhang H. et al., 2021). Furthermore, Yadav et al. proved that miR-5096 regulates ferroptosis by targeting and downregulation of SLC7A11. When miR-5096 is expressed ectopically, it enhances the accumulation of iron and oxidation of GSH, which leads to ferroptosis. Additionally, they discovered that miR-5096 promoted the expression of EMT makers *in vitro* and inhibited the propensity for metastasis in MDA-MB-231 cells *in vivo* (Yadav et al., 2021). Liang et al. has identified that circular RNA circ-ABC10 shows high expression in breast cancer, compared with peripheral non-cancerous zones. The evidence was strengthened by the discovery that miR-1271 targets circABC10 by bioinformatics analysis and luciferase reporter assay. Remarkably, the reduction of circ-ABC10 triggered cell apoptosis enhancement but there is no direct evidence linking this to ferroptosis (Liang et al., 2017). All of these provide theoretical justifications for how ncRNAs control ferroptosis in TNBC cells.

**FIGURE 4:**
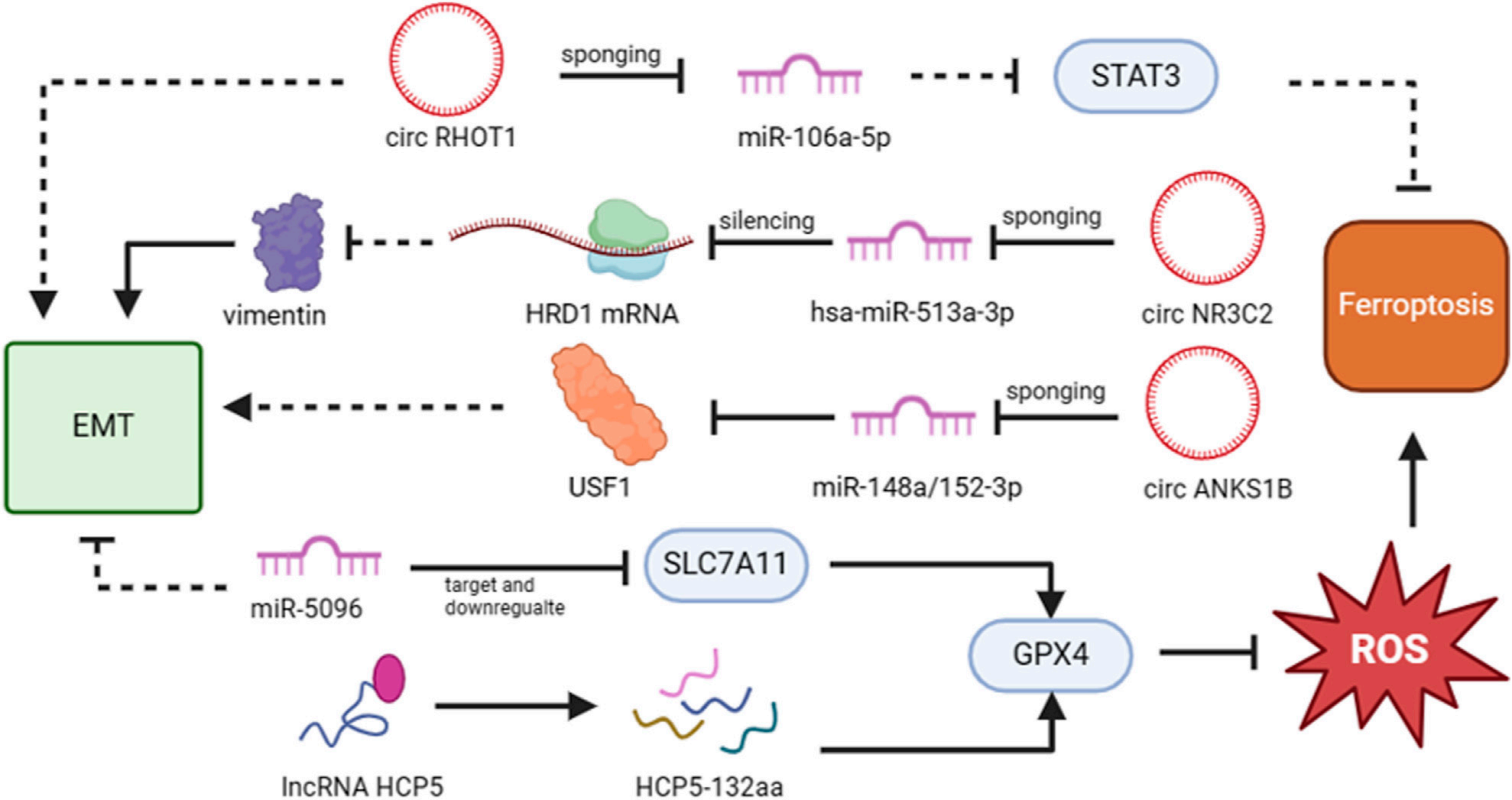
The functions of non-coding RNAs that target ferroptosis and EMT in TNBC metastasis. Non-coding RNAs that target EMT/ferroptosis-related pathways or proteins can have either a favorable or unfavorable impact on the metastasis of TNBC cells. The figure was created using BioRender.com.

Additionally, ncRNAs can regulate the metastasis of TNBC by EMT program. Has_circ_001783 was high-level expressed in breast cancer cells, and is obviously upregulated in TNBC cells in the contrast of other two types of breast cancers. When the has_circ_001783 expression level was knocked down, it would inhibit the proliferation and metastasis of TNBC cells. Also, it is confirmed that miR-200c-3p is negatively related with the sponging target of has_circ_001783. Namely, low expression of has_circ_001783 triggered the expression of miR-200c-3p. Furthermore, a decrease in has_circ_001783 expression prevents the EMT regulators ZEB1, ZEB2, and ETS1 from regulating TNBC metastasis (Liu et al., 2019). In addition, EIF6-224aa, transformed from circ-EIF6, directly binds to MYH9 protein and inhibits its degradation. Then wnt/β-catenin pathway was triggered, facilitating circ-EIF6 to promote the proliferation and metastasis of TNBC (Li Y. et al., 2022). Moreover, long non-coding RNAs are also frequently used to diagnose and prognosticate the tumor development. Certainly, there are several lncRNAs regulate the development of TNBC (Kaushik et al., 2020). In addition to the relevant downstream target or pathway acting as an upstream regulator, lncRNA can also be targeted by upstream genes. For instance, TUFTI promotes the progression of TNBC by upregulating the expression of the lncRNA DANCR via the miR-874-3p-SOX2 pathway (Wu et al., 2020) (Figure 4; Table 1).

**TABLE 1:** The role of non-coding-RNA in breast cancer progression.

Non-coding RNA	Abundance	Target/mechanism	Function on BC	Ref.
circ-ABC10	High	-miR-1271	-Promote proliferation -Suppress apoptosis	Liang et al. (2017)
has_circ_001783	High	-miR-200c-3p	-Promote growth -Promote metastasis	Liu et al. (2019)
circRHOT1	High	-miR-106a-5p/STAT3 axis	-Inhibit ferroptosis	Zhang et al. (2021b)
miR-5096	Low	-SLC7A11/xCT	-Inhibit EMT -Inhibit ferroptosis	Yadav et al. (2021)
miR-183-5p	High	-PDCD4	-Promote proliferation	Cheng et al. (2016), Wang et al. (2019a)
miR-802	Low	-FoxM1	-Inhibit proliferation	Yuan and Wang (2015)
circ-EIF6	High	-wnt/β-catenin pathway	-Promote EMT	Li et al. (2022c)
circ-TRIO	High	-miR-432-5p/CCDC58 axis	-Promote proliferation -Promote metastasis	Wang et al. (2022b)
circAHAKI	Low	-miR-421 and RASA1	-Inhibit proliferation	Xiao et al. (2019)
miR-199a-5p	Low	-PIK3CD	-Inhibit EMT	Chen et al. (2016)
miR-204	High	-CDC42, CDH1, CDH2, NTRK2, SNAI1, SNAI2, STAT3, TWIST1	-Inhibit self-renewal -Inhibit EMT	Rahimi et al. (2019)
miR-200c	Low	-CFL2, FN1, MAPK9, MUC1, RHOA, ROCK2, SNAIL1, ZEB1/2	-Inhibit self-renewal -Inhibit EMT	Rahimi et al. (2019)
miR-10b	Low	-CDH1, CDH2, MYC, SNAIL1, SALL4, SMAD4, TWIST1	-Inhibit self-renewal -Inhibit EMT	Rahimi et al. (2019)
miR-34a	Low	-IL-6, CDH1, FOX2, SMAD4, SNAI1, STAT3, ZEB1	-Inhibit EMT	Pan et al. (2021), Weng et al. (2019), Rahimi et al. (2019)
lncRNA SOX2OT	High	-miR-942-5p -Activation PI3K/Akt pathway	-Promote EMT	Zhang et al. (2022)
lncRNA H19	High	-p53/TNFAIP8 pathway -Akt pathway	-Promote EMT -inhibits the sensitivity of paclitaxel	Li et al. (2020c), Han et al. (2018)
lncRNA PTCSC3	Low	-Downregulation lncRNA H19	-Inhibit metastasis	Wang et al. (2019b)
lncRNA DRHC	Low	-Downregulation lncRNA HOTAIR	-Inhibit proliferation	Yu et al. (2019)
lncRNA GAS5	Low	-miR-196a-5p -FOXO1/PI3K/AKT pathway	-Inhibit proliferation -Promote apoptosis	Li et al. (2018)
lncRNA XIST	Low	-miR-454	-Inhibit EMT	Li et al. (2020d)
lncEPCAM	High	—	-Promote metastasis -Promote Proliferation	Barton et al. (2019)

## 6 The therapy for TNBC

For years, chemotherapy has been the primary option for TNBC patients who have lower survival ratio and worst prognosis because of its high metastasis incidence and lack of effective post-recurrence treatment (Adinew et al., 2021; So et al., 2022). The likelihood of TNBC’s response to immunotherapy is heightened by several characteristics of TNBC cells, such as augmented tumor-infiltrating lymphocytes and augmented expression of programmed death-ligand 1 (PD-L1) (Keenan and Tolaney, 2020). Unfortunately, it has been discovered that the PD-L1 inhibitor monotherapies avelumab and atezolizumab are insufficient for treating metastatic triple negative breast cancer (mTNBC). However, early-stage TNBC patients have shown evidence of the initial efficacy of using ICIs in combination with chemotherapy (Keenan and Tolaney, 2020).

Additionally, data demonstrates an apparent benefit of chemotherapy when paired with neoadjuvant, adjuvant, and metastatic settings (Bianchini et al., 2016). Anti-programmed death-ligand 1 (anti-PD-L1) antibodies applying to the treatment of PD-L1-positive mTNBC, can improve progression-free survival (PFS) and overall survival (OS) of patients (Loibl et al., 2022). A randomized controlled trial (RCT) revealed that based on anthracycline/taxane neoadjuvant chemotherapy (NACT), added with durvalumab (the PD-L1 inhibitor), increased the pathological complete response (pCR) rate in TNBC patients (Loibl et al., 2019). Another RCT found that giving durvalumab to TNBC patients as a maintenance therapy improved their overall survival, particularly in those with PD-L1+ TNBC (Bachelot et al., 2021).

## 7 Targeting non-coding RNAs/EMT/ferroptosis axis for TNBC treatment

As described above, the ncRNA, EMT, and ferroptosis plays significant roles in TNBC progression. Here, we hope to find a genuine treatment for TNBC patients, starting with non-coding RNA targeting ferroptosis or/and EMT. There is evidence that TNBC cells are sensitive to ferroptosis, and this suggests a potential therapeutic option for TNBC that involves promoting this type of non-apoptotic cell death (Sun L. L. et al., 2021). Obvious study demonstrated that metformin regulated ferroptosis in MDA-MB-231 cells. Its mechanism is that metformin upregulated the expression of miR-324-3p, which directly binds to the 3′UTR of GPX4 to downregulate its expression. And the metformin plays the role of anti-cancer by suppressing EMT and metastasis features (Hou et al., 2021). Moreover, in MDA-MB-468 TNBC cells, metformin induced many associated enzymes for glucose metabolism, suggesting that metformin may be used in the treatment of TNBC (Samuel et al., 2019). Local anesthetic lidocaine downregulated SLC7A11 to induce ferroptosis by targeting miR-382-5p and promoting its expression in breast cancer cells (Sun D. et al., 2021). In addition, lidocaine is cytotoxic to the MDA-MB-231 and BT-474 breast cancer cell lines, and researchers found that in these two cell types, lidocaine reduced ROS without the discussion of ferroptosis (Chen et al., 2022). Consequently, the modulation of non-coding RNA expression by drugs exerts regulatory control over both EMT and/or ferroptosis, thereby influencing a profound impact on the progression of TNBC.

Considering on another perspective, can ncRNAs modulate treatment resistance in TNBC by affecting EMT and/or ferroptosis? Paclitaxel (PTX), an anti-tumor drug, is a crucial part of the chemotherapeutic treatment for TNBC. Downregulation of circWAC, which targets miR-142, increased the sensitivity to PTX in TNBC cells (Wang L. et al., 2021). The utilization of crizotinib, an anti-ROS1 agent, has demonstrated resistance in the treatment of triple-negative breast cancer (TNBC). Subsequent investigation revealed that TNBC cells resistant to crizotinib exhibited elevated levels of FAK expression. By combining the FAK inhibitor IN10018 with crizotinib, there was an increase in ROS levels, upregulation of p53 expression, and downregulation of SCL7A11 and GPX4. Ultimately, this led to ferroptosis and exerted potent anti-tumor effects (Tan et al., 2024). Evidence shows that the as-obtained Fe3O4@PCBMA-SIM nanoparticles show more cytotoxicity to MAD-MB-231. Since SIM can suppress the expression of HMGCR, leading to the downregulation of the mevalonate (MVA) pathway and GPX4, ultimately inducing ferroptosis (Yao X. et al., 2021). Moreover, the combined use of CPT/Fe@PDA-FA10 and laser treatment demonstrates remarkable efficacy in inducing apoptosis/iron death/photothermal therapy in drug-resistant TNBC cells. Remarkably, this approach exhibits negligible adverse effects on major tissues and organs (Cai et al., 2023). The construction of tumor-targeted combinatorial nanoparticles can effectively induce ferroptosis, thereby overcoming tumor resistance in triple-negative breast cancer.

The improvement of tumor therapy efficacy is achieved by employing nano-prodrugs for targeted delivery of drugs with different mechanisms of action to the tumor microenvironment (Chen et al., 2017; Wu et al., 2023). Actively targeted small molecule self-assembled nano-prodrug are employed for the co-delivery of chemotherapeutic agents (CPT), ferrocene (Fc), and GPX4 inhibitors (RSL3). Upon reaching the tumor microenvironment, this nano-prodrug undergoes GSH-mediated degradation to facilitate the release of encapsulated substances, thereby augmenting the efficacy of chemotherapeutic agents through induction of cellular iron death and apoptosis (Chen et al., 2023).

Non-coding RNAs have essential functions in the drug-therapeutic resistance of TNBC; miRNA and lncRNA, in particular, play significant roles in chemoresistance by regulating a variety of genes and related pathways (Ferrari et al., 2022). MCT-1, a poor prognosis marker of invasive breast cancer, further induces STAT3 activation and the expression of snail, ZEB1, and N-cadherin to accelerate EMT through the IL-6 pathway. When treated with an MCT-1 antagonist, the miR-34a can reduce IL-6 expression, which increases the therapeutic effectiveness of TNBC (Weng et al., 2019). Repression of miR-155-5p expression in TNBC cells attenuates the resistance of cetuximab through the induction of cell apoptosis and pyroptosis (Xu et al., 2021). However, the impact of reducing miR-155-5p levels on drug resistance through ferroptosis remains to be elucidated. The overexpression of miR-33a-5p resulted in the inhibition of eIF5A2 expression and attenuated the EMT process induced by doxorubicin (Dox), ultimately confirming its ability to enhance the sensitivity of TNBC cells to Dox (Guan et al., 2019). LncRNA DLX6-AS1 prompts EMT and cisplatin resistance in TNBC scenario through miR-199b-5p/PXN pathway (Du et al., 2020). However, it did not clearly elucidate the association between EMT and cisplatin resistance within this signaling pathway.

## 8 Conclusions and perspectives

Triple-negative breast cancer is the most aggressive type of breast cancer, which exhibits poor prognosis and high recurrence (Won and Spruck, 2020; Vagia et al., 2020). The treatments that are currently available are insufficient to treat TNBC tumors that are inoperable or recurring. A large unmet need still exists for the development of innovative therapeutics for TNBC, particularly in advanced stages, to inhibit the metastasis of TNBC, and improve the prognosis of breast cancer patients. Epithelial-mesenchymal transition and ferroptosis are major biological processes in cancer metastasis, and there are various signaling pathways and non-coding RNAs play a complex role in both ferroptosis and the EMT processes.

In this review, we briefly introduced the discovery and hallmarks of ferroptosis, as well as the process of EMT, and focused on summarizing the roles of ferroptosis and EMT in TNBC progression. Moreover, we also discussed how ncRNAs regulate ferroptosis and EMT, as well as the functional roles in regulation metastasis of breast cancer. However, there remains a few unresolved inquiries. 1) To date, only a few studies have elucidated the crosstalk between ferroptosis and EMT in neoplastic processes, including Panc-1 cancer stem-like cells, lung adenocarcinoma, and breast cancer (Yao J. et al., 2021; Chen X. et al., 2021; Müller et al., 2020; Peng et al., 2019). Further investigation is necessary to elucidate the underlying mechanism linking ferroptosis and EMT in the context of TNBC. 2) Numerous targeted non-coding RNAs have been identified to play pivotal roles in the processes of EMT or ferroptosis. However, there is still limited literature available that directly demonstrates the presence of non-coding RNAs regulating both EMT and ferroptosis in TNBC.

Taken together, in this review, we collected the link between ferroptosis, EMT, and ncRNAs in the metastasis processes of TNBC. While the further study still needs to be done to explore the underlying mechanisms of the ncRNAs/ferroptosis/EMT axis in TNBC progression. This interplay may facilitate the development of potential therapeutic approaches to eliminate cancer cells, and provide a comprehensive understanding of the metastasis mechanism of TNBC to identify novel treatment strategies.
